# Conservative management of spontaneous left iliac vein rupture in May-Thurner syndrome

**DOI:** 10.1016/j.jvscit.2025.101934

**Published:** 2025-07-22

**Authors:** Nam Ho, Thu Thi Nguyen, Vinh Huy Tuan Pham, Trung Dinh Ngo

**Affiliations:** a Surgical and Transplant Intensive Care Unit, Military Central Hospital 108, Hanoi, Vietnam; b VinUniversity, Hanoi, Vietnam

**Keywords:** Iliac vein, Venous thrombosis, May-Thurner syndrome, Retroperitoneal hemorrhage, Vascular diseases

## Abstract

We report two rare cases of spontaneous left iliac vein rupture associated with May-Thurner syndrome. A 60-year-old patient presented with acute lower abdominal pain and was diagnosed with a retroperitoneal hematoma and left iliac vein thrombosis. She was managed conservatively with anticoagulation and inferior vena cava (IVC) filter placement, resulting in complete thrombus resolution within 2 months. A second patient, aged 65 years, presented in hemorrhagic shock with extensive deep vein thrombosis of the left lower limb. After resuscitation and transfusion, she underwent IVC filter placement and anticoagulation, with full thrombus resolution confirmed at the 3-month follow-up. These cases highlight the diagnostic challenges of spontaneous iliac vein rupture and the importance of early recognition. Conservative management with anticoagulation and IVC filter placement may be a safe and effective alternative to surgery in stable patients.

Spontaneous iliac vein rupture (SIVR) is a rare but life-threatening vascular emergency, often presenting with retroperitoneal hemorrhage and hemodynamic instability.^1^ The etiology remains unclear, but is frequently linked to predisposing factors such as May-Thurner syndrome (MTS), venous hypertension, or connective tissue disorders.^2^ Clinical manifestations are typically acute and nonspecific, including abdominal or pelvic pain, hypotension, tachycardia, and signs of internal bleeding.^3^ Owing to its rarity and variable presentation, SIVR poses significant diagnostic challenges. Prompt recognition and tailored management are essential to prevent fatal outcomes.^4^ We report two rare cases of spontaneous left iliac vein rupture associated with MTS, focusing on clinical presentation, diagnostic workup, and successful conservative treatment strategies. Informed consent was obtained from the patients for publication of this report.

## Case reports

### Patient 1

A 60-year-old woman with no history of deep vein thrombosis (DVT), hypertension, trauma, or systemic disease presented with progressive aching pain and numbness in the left leg for 4 days, followed by fatigue, dizziness, cold sweats, and left iliac fossa pain 3 days before admission. Initially treated at a local hospital without improvement, she was transferred to our center for further evaluation. No relevant medical, family, or psychosocial history was reported.

On admission, vital signs were stable: blood pressure 105/60 mm Hg, heart rate of 90 bpm, respiratory rate of 20 breaths per minute, and oxygen saturation of 99% on 3 L/min oxygen. The patient was alert but fatigued, with pale skin and mucous membranes. Physical examination revealed a swollen, tense, and tender left leg. Neurological status was intact.

Laboratory tests showed anemia with red blood cell (RBC) count of 2.69 × 10^12^/L, hemoglobin of 85 g/L, hematocrit of 0.214, and markedly elevated D-dimer (5516 ng/mL). Coagulation parameters and platelet count were normal. Other blood chemistry tests were unremarkable.

**Fig 1:**
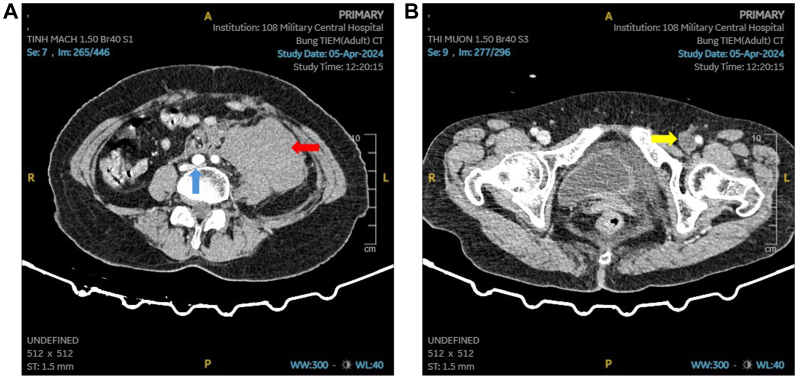
**(A)** A transverse computed tomography (CT) angiogram of the abdomen and pelvis demonstrated a large left-sided retroperitoneal hematoma consistent with extensive hemorrhage, without evidence of active bleeding (*red arrow*). The left common iliac vein is compressed between the right common iliac artery and the lumbar spine, consistent with May-Thurner syndrome (MTS) (*blue arrow*). **(B)** Complete occlusion of the external iliac veins (*yellow arrow*).

A computed tomography (CT) scan revealed a large retroperitoneal hematoma and complete occlusion of the left common, internal, and external iliac veins, consistent with MTS owing to compression of the left common iliac vein by the right common iliac artery and vertebral body. No contrast extravasation was noted. The aorta, iliac arteries, and abdominal organs were normal (Fig 1, *A* and *B*; Supplementary Video 1, online only). Color Doppler ultrasound examination confirmed extensive DVT in the left lower limb.

The diagnosis was spontaneous left iliac vein rupture with retroperitoneal hemorrhage secondary to MTS. On hospital day 2, an inferior vena cava (IVC) filter was inserted, and anticoagulation with subcutaneous enoxaparin 60 mg was initiated. After 3 days, the patient's symptoms improved significantly, with reduced leg edema and resolution of abdominal pain. She was transitioned to oral dabigatran 300 mg/d.

By day 9, the patient ambulated normally without leg pain or tenderness. Doppler ultrasound examination showed residual thrombosis in the left external iliac, common femoral, and deep femoral veins, but anemia had improved (RBC count of 3.59 × 10^12^/L, hemoglobin of 108 g/L). She was discharged after 2 weeks, with plans for IVC filter removal after 3 weeks. At the 2-month follow-up, ultrasound examination confirmed complete thrombus resolution.

### Patient 2

A 65-year-old woman with a history of depression treated with olanzapine 10 mg/d presented with a 2-day history of rapidly worsening left iliac fossa pain. She denied fever and had normal bowel movements. On the day before admission, she became pale, fatigued, and experienced syncope during toileting, prompting transfer to the intensive care unit.

On presentation, the patient was lethargic with pale mucous membranes. Vital signs showed hypotension (blood pressure of 90/60 mm Hg) maintained with noradrenaline at 0.1 μg/kg/min, tachycardia (heart rate of 110 bpm), respiratory rate 20 breaths per minute, and oxygen saturation 98% on room air. Physical examination revealed a warm, tender left iliac fossa abdomen and tender erythematous swelling of the entire left leg from the ankle to the thigh.

Laboratory tests revealed anemia (RBC count of 2.5 × 10^12^/L, hemoglobin of 75 g/L, hematocrit of 0.20) and a normal platelet count (250 × 10^9^/L). Coagulation studies showed a prothrombin time of 94%, activated partial thromboplastin time of 25 seconds, fibrinogen of 3.6 g/L, and markedly elevated D-dimer (>7000 ng/mL). Arterial blood gas analysis indicated metabolic acidosis (pH 7.2, partial pressure of oxygen of 80 mm Hg, partial pressure of carbon dioxide of 31 mm Hg, HCO_3_^−^ of 21 mmol/L, base excess of −5 mmol/L, and lactate of 3.6 mmol/L).

**Fig 2:**
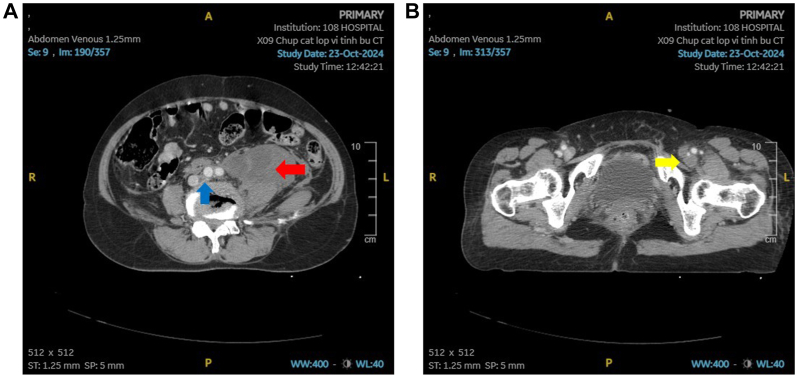
**(A)** Transverse computed tomography (CT) angiography of the abdomen and pelvis demonstrates a large retroperitoneal hematoma on the left (*red arrow*) compressing the left common iliac vein between the right common iliac artery and the vertebral body, resulting in complete occlusion (*blue arrow*). **(B)** Complete occlusion of the external iliac veins (*yellow arrow*).

Abdominal ultrasound examination revealed a heterogeneous fluid collection (114 × 50 mm) along the left paracolic gutter to the left iliac fossa. Doppler ultrasound examination of the left lower limb showed extensive thrombosis involving the iliac, femoral, superficial femoral, popliteal, anterior tibial, and posterior tibial veins. CT of the abdomen and pelvis demonstrated fluid accumulation in the left retroperitoneal space extending from the pelvis to the left kidney anterior to the psoas muscle, with hyperdense fluid (69 Hounsfield units), but no active bleeding. Complete occlusion of the left common, internal, and external iliac veins was consistent with MTS owing to compression of the left common iliac vein by the right common iliac artery and vertebral body (Fig 2, *A* and *B*; Supplementary Video 2, online only).

The diagnosis was hemorrhagic shock secondary to spontaneous rupture of the left iliac vein associated with MTS. Initial management included transfusion of 1000 mL packed RBCs, vasopressor support with noradrenaline, and placement of an IVC filter. The patient stabilized within 24 hours. Anticoagulation with oral dabigatran 300 mg/d was started on hospital day 3.

The patient improved and was discharged after 1 week. The IVC filter was removed 2 weeks later. Follow-up ultrasound examination showed persistent thrombus, so anticoagulation was continued for 3 months. Complete resolution of DVT in the left lower limb was confirmed on follow-up.

Ethical approval was not required for this case report. Written informed consent for publication was obtained from the patients.

## Discussion

SIVR is a rare but potentially fatal condition, frequently associated with MTS.^2^ We report two cases involving Vietnamese females aged 60 and 65 years, both presenting with left-sided retroperitoneal hematoma and DVT. These cases highlight the diagnostic complexity and therapeutic challenges of SIVR, particularly in the context of limited resources.

SIVR predominantly affects women (84.2%) at a mean age of 61 years and most commonly involves the left iliac vein (96.1%), often in association with DVT. Presenting symptoms typically include abdominal or back pain, leg swelling, and hemorrhagic shock.^5^ The underlying pathophysiology remains unclear but may involve mechanical compression (MTS), inflammation, hormonal changes, or increased intra-abdominal venous pressure.^6^ Diagnosis requires prompt imaging using venous duplex ultrasound examination, contrast-enhanced CT scan, or catheter-based venography to identify vessel rupture, thrombosis, or venous compression.^7^

MTS, first described in 1957, results from compression of the left common iliac vein by the right common iliac artery and the lumbar spine, causing endothelial damage to the vein, leading to fibrosis or intimal spurs, which result in stenosis and occlusion of the vein.^8^^,^^9^ It is found in up to 24% of the population, with a greater prevalence in women.^10^ MTS is often overlooked owing to more common risk factors for DVT, such as oral contraceptives, recent pregnancy, or thrombophilia, leading to recurrent DVT and post-thrombotic syndrome.^11^ This syndrome accounts for approximately 2% to 5% of DVT cases with a prevalence twice as high in women as in men.^8^^,^^12^ Recent data suggest that MTS may underlie up to 41.8% of SIVR cases.^1^

Management strategies for SIVR include surgical, endovascular, or conservative approaches. In a review of 55 cases, open surgery (eg, ligation, repair, or bypass) was used in 54.5% of the cases, with a mortality rate of 20.0% and a complication rate of 63.3%.^1^ These rates align with past reviews reporting mortality rates between 23.8% and 29.0%.^6^ In contrast, endovascular treatment, such as stenting, was used in 21.8% of cases, demonstrating lower complication rates (33.3%) compared with surgical interventions (63.3%). Hybrid approaches that combined surgical and endovascular methods accounted for 3.6% of cases.^1^ Additionally, conservative management, which involves stabilizing measures with or without anticoagulation, was used in 9.1% of cases and demonstrated viability, particularly in selected patients. Notably, 7.3% of patients received no intervention owing to postpartum recovery or death before planned treatment. The presence of MTS influenced treatment patterns. Among patients with MTS, endovascular treatment was the most common approach (39.1%), followed by surgery-only treatment (21.7%). For non-MTS patients, surgery remained predominant (78.1%). Interestingly, nonsurgical treatment options increased from 17.9% before 2012 to 74.1% after, reflecting a shift toward less invasive strategies owing to the higher risks associated with surgery.^13^ Despite potential reporting biases favoring favorable outcomes, this trend highlights growing reliance on endovascular and conservative approaches for managing SIVR effectively.^1^

In the context of the presented cases, similarities with previously published reports include demographic and clinical features such as left-sided retroperitoneal hematomas and concurrent DVT. However, notable differences in management exist. Historically, surgical interventions, such as ligation or repair, have been the predominant treatment strategy, used in 54.5% of cases in the study by Skeik et al. (2023).^1^ Surgery has been associated with a high mortality rate of 20.0% and complication rate of 63.3%, as shown in reviews by Hosn et al. (2016).^14^ Endovascular treatments, such as stenting, used in 21.8% of cases, demonstrate lower complication rates (33.3%) but require advanced resources and expertise. In contrast, the presented cases were managed conservatively with anticoagulation and IVC filter placement. This approach reflects a growing trend toward less invasive strategies, particularly for hemodynamically stable patients without active bleeding. Although case 2 initially presented in hemorrhagic shock, she stabilized within 24 hours after transfusion and low-dose vasopressor support. Crucially, a contrast-enhanced CT scan showed no active extravasation, and her condition remained stable thereafter. Based on these findings and limited access to endovascular intervention, conservative treatment with anticoagulation and IVC filter placement was deemed appropriate and successful. In the study by Kooiman et al. (2023),^5^ conservative management, used in 9.1% of cases, avoids risks such as infection and venous scarring, making it a viable option in resource-limited settings.

The differences in management have significant implications for treatment strategies. In low-resource settings, where access to surgical or endovascular interventions may be limited, conservative management provides a feasible alternative. At our center, endovascular stenting was not available at the time of patient presentation. Although staged correction of MTS is ideal, resource limitations precluded this approach. Patients remained under follow-up, and no clinical recurrence has been observed to date. Retrievable IVC filters were used as a precautionary measure to prevent pulmonary embolism in the context of extensive iliac thrombosis. We elected to place filters selectively in both cases. Currently, there is no consensus on the optimal duration of anticoagulation for conservatively managed SIVR. In our cases, oral dabigatran was administered for 3 months, following standard guidelines for provoked DVT.

Further studies are needed to define the optimal duration and assess recurrence risks. The use of direct oral anticoagulants such as dabigatran in the presented cases highlights their potential advantages over warfarin, including ease of use and better compliance.^15^ However, the decision to adopt conservative treatment must be guided by careful patient selection, ensuring that patients are hemodynamically stable and free from active bleeding. These findings emphasize the importance of prompt imaging to assess stability and confirm the absence of active extravasation.^1^

## Conclusions

The key takeaway from these cases lies in the importance of understanding the interplay between pathophysiology, presentation, and resource availability in guiding treatment decisions. For stable patients without active bleeding, conservative management with anticoagulation represents a viable and safe alternative to surgery or endovascular techniques. These findings underscore the need for further research into patient selection criteria, optimal anticoagulation regimens, and long-term outcomes to refine treatment guidelines for this rare but challenging condition.

## Availability of Data and Material

All data related to this case report are included in the manuscript. Additional clinical details can be made available by the corresponding author upon reasonable request, ensuring compliance with patient confidentiality regulations.

## Funding

None.

## Disclosures

None.
